# Supplementary material for: Are carbon emissions trading and green financial instruments synergistic? -Comprehensive quantitative research based on content analysis
Source: PLoS One. 2024 Mar 7;19(3):e0298601. doi: 10.1371/journal.pone.0298601 (PMC10919601; doi:10.1371/journal.pone.0298601)
Supplement: S1 Data — (ZIP) [file pone.0298601.s001.zip › Data/地级市Z_F工作报告.zip!/╒π╜¡/║╝╓▌╩╨/└┤╫╘╣1⁄2╓┌║┼í╛┬φ┐╦╩2╛▌═°í┐_2017.docx]

各位代表：

　　现在，我代表市人民政府向大会报告工作，请予审议，并请市政协委员和其他列席人员提出意见。

**一、2016年和本届政府工作回顾**

　　2016年，我们全面贯彻党的十八大和十八届三中、四中、五中、六中全会精神，深入贯彻习近平总书记系列重要讲话和对杭州工作重要指示批示精神，在党中央国务院、省委省政府和市委的领导下，以服务保障G20杭州峰会为圆心，推动经济社会持续稳定健康发展，本届政府工作圆满收官，“十三五”发展精彩开局。过去一年，我们主要抓了以下工作：

　　一是举全市之力服务保障G20杭州峰会。认真贯彻习近平总书记重要指示精神，以“最高标准、最快速度、最实作风、最佳效果”为要求，全力做好场馆改造、会议服务、平安护航、氛围营造以及6大类605个环境整治提升项目等峰会各项服务保障工作，圆满实现了“四个满意”目标，得到习近平总书记的充分肯定、高度赞扬。动员全市广大干部群众积极参与“服务G20，人人都是东道主”主题活动，识大体、顾大局、讲奉献，弘扬“精致和谐、大气开放”的城市人文精神，树立“诚信包容、文明友善”的市民形象，向世界展现了杭州历史与现实交汇的独特韵味，为成功举办峰会贡献了杭州的智慧和力量。抓住“后峰会、前亚运”窗口期，以打造“四大个性特色”、形成“四大基础支撑”为重点，加快城市国际化步伐，入选全球国际会议目的地百强城市、全球52个最值得到访的旅游目的地。

　　二是持续发力促进经济提质增效。全市生产总值达到11050.49亿元，增长9.5%，增幅居全国副省级城市第一；一般公共预算收入达到1402.38亿元，增长13.2%，总量居省会城市第一。完成固定资产投资5842.42亿元，增长5.1%，货物出口3313.8亿元，增长6.7%；其中跨境电商出口60.6亿美元，增长166.7%。全社会消费品零售总额5176.2亿元，增长10.5%；网络零售额3445.65亿元，增长28.6%。经济结构持续优化，三产增加值占全市生产总值比重首次超过60%。扎实推进“一号工程”，制定出台《中国制造2025杭州行动纲要》，规上高新技术产业、装备制造业、战略性新兴产业增加值分别增长12.5%、14.6%和11.6%；信息经济增加值达到2688亿元，增长22.8%。成功举办西湖国际博览会、中国国际动漫节、中国（杭州）国际电子商务博览会、杭州文化创意产业博览会、杭州云栖大会；成功举办首届世界工业设计大会，会址永久落户杭州。扎实推进“三去一降一补”，淘汰改造落后产能企业（生产线）213家（条），整治低小散企业（作坊）874家；出台“扶持实体经济32条”，为企业减负300亿元以上；做好房地产市场调控，全年商品房销售面积2326.69万平方米。

　　三是扎实推动改革开放创新举措落地。深化“放管服”改革，“五证合一”商事登记办法向全国推广。稳步推进户籍制度改革、不动产统一登记制度改革，深化国资国企改革、农村产权制度改革、村（社区）集体经济股份制改革，“三位一体”农民合作经济组织较快发展。

　　积极推进人民警察职业序列改革试点工作。出台网络预约出租车和私人小客车合乘管理实施细则。发布全国首个跨境电商发展指数，跨境电商上线企业超过6000家，“六体系两平台”跨境电商模式向全国推广。浙商回归资金、到位内资、实际利用外资分别增长7.5%、11.8%和1.4%；新增上市企业17家、新三板挂牌企业192家；新设各类市场主体16.93万户，增长25.5%。推进国家自主创新示范区建设，实施科技企业“双倍增”行动计划，新增国家重点扶持高新技术企业516家、省科技型中小企业1521家。未来科技城、阿里巴巴集团入选国家首批“双创示范基地”。制定出台城西科创大走廊、钱塘江金融港湾建设规划和支持政策。制定出台“人才意见22条”，新增“国千”人才19名、“省千”人才54名。成立浙江西湖高等研究院、浙江工程师学院，新型高校建设取得突破。杭州都市圈和都市区建设扎实推进。山海协作、对口支援和扶贫协作工作进一步加强。

　　四是大力推进城市建设和环境治理。城市总体规划（修订）批准实施，市政府驻地顺利迁址，钱江新城、钱江世纪城发展进入新阶段。杭黄铁路加快建设，杭富、杭临城际铁路试验段开工建设，富春江船闸开通运行。地铁二期工程加快建设，三期规划获国家批准。萧山机场路、东湖快速路、紫之快速路等一批城市道路建成通车，主城区新建停车泊位5.1万个。交通综合治堵和公交都市创建工作持续深化，城市交通拥堵状况有所缓解。入选全国地下综合管廊试点城市，开发利用地下空间723万平方米。湘湖综合保护工程三期全面建成。持续推进“五水共治”，夺得全省治水工作最高奖“大禹鼎”；新增污水管网269公里，完成7座污水处理厂一级A提标改造，完成160个行政村生活污水治理。市控以上断面水质达标率85.1%，钱塘江杭州段干流均达到或优于Ⅲ类水质。千岛湖配供水工程顺利推进，闲林水库下闸蓄水，祥符水厂一期改造提前完成。扎实推进“五气共治”，三区四县（市）中心城区建成“无燃煤区”，主城区节能及新能源公交车占比达95.78%，建筑工地扬尘监管全覆盖；空气优良天数比上年增加18天。积极推进“五废共治”，九峰环境能源项目扎实推进，市第二固废处置中心投用，工业固废、生活垃圾、医疗废物无害化处理工作进一步加强。深入推进“三改一拆”，完成“三改”3349.7万平方米，拆除违法建筑2182.4万平方米，腾出土地2.67万亩，拆后利用2.09万亩；打响城中村改造和小城镇环境综合整治攻坚战，23个村完成全面改造。深化美丽乡村建设，开展杭派民居示范创建村12个。扎实推进节能减排，单位生产总值能耗预计下降6%以上。进一步加大环境执法力度，立案查处环保违法案件1972件。

　　五是着力加强民生和社会保障。全市一般公共预算中民生支出占比达75%以上，年度10件民生实事项目全面完成。城镇和农村居民年人均可支配收入分别增长8%和8.5%；基本养老、医疗保险参保率分别达95%和98%以上。开工建设保障性住房7.61万套，竣工5.86万套。全市竣工新建中小学校（幼儿园）57所，省义务教育标准化学校覆盖率达93%以上，主城区名校集团化覆盖率超过81%。浙大城市学院怀卡托大学联合学院获准建设。医养护签约服务政策实现城乡全覆盖，5家市属三甲医院托管18家县级医院。首批省级养老服务业综合改革试点正式启动。公共文化服务标准化工作加快推进，群众精神文化生活日益丰富。平安杭州建设深入推进，连续三年实现命案全破，安全生产主要指标继续好转，食品安全的群众满意度有较大提高。国防动员和后备力量建设加强，荣获全国人防工作先进城市，实现全国双拥模范城“七连冠”。外事、港澳台侨事务、民族宗教、双拥优抚、人口计生、妇女儿童、残疾人、老龄、关心下一代、地方志、气象、红十字、慈善、机关事务管理等工作取得新进展。

　　各位代表！去年全市主要目标任务顺利完成，为本届政府工作划上了圆满句号。本届政府任期的五年，是杭州发展史上极不平凡、印上浓墨重彩、具有重大转折意义的五年。我们经受了重大考验、实现了跨越发展，经济实力大幅增强、城乡面貌日新月异，在全省的龙头地位全面巩固、在全国的战略地位显著提升、在国际上的影响力明显增强。

　　——这五年，城市地位在落实国家战略中实现大跃升。我们成功举办G20杭州峰会，获得2022年亚运会、2018年世界短池游泳锦标赛和世界游泳大会承办权，成为2017年全国学生运动会举办城市，获得航空口岸144小时过境免签政策，城市知名度和影响力大幅提升；批准设立全国首个跨境电商综试区，批准建设国家自主创新示范区，成功创建和扎实推进国家生态文明先行示范区、杭州都市经济圈转型升级综合改革、国家创新型城市、中国软件名城、知识产权示范城市、服务贸易创新发展、中国快递示范城市等国家试点，对杭州发展起到了重大牵引作用。

　　——这五年，经济实力在加快转型升级中跨上大台阶。我们主动适应把握新常态，大力推进结构调整，经济实现又好又快发展。全市生产总值从2011年的7037.28亿元提升到2016年的11050.49亿元，居全国经济总量超万亿元城市第十位，年均增长9.0%，占全省经济总量比重上升至23.8%。三次产业结构从3.4∶46.6∶50.0优化为2.8∶36.0∶61.2。信息经济引领发展，占全市生产总值比重提高到24.3%，对全市经济增长贡献率超过50%；高新技术产业增加值年均增长11.4%，占全市生产总值比重提高到12.4%；文创、金融、旅游等特色主导产业发展迅速。一般公共预算收入从785.15亿元提升到1402.38亿元，年均增长12.3%；2016年税收占一般公共预算收入比重达到91.9%。在经济总量较快增长的同时，质量效益得到明显提升。

　　——这五年，创新能力在深化改革开放中得到大增强。我们积极推进区域管理体制创新，大江东管理体制基本理顺，萧山、余杭、富阳与主城区融合发展深度推进，主城区优质公共服务和要素资源加速扩展，一体化效应逐步显现。行政审批、商事登记制度改革成效显著，政府性投资项目和社会投资项目实行以区为主审批管理的制度；市场主体从52.44万户发展到86.17万户，年均增长10.4%。杭州高新区开发进入国家世界一流高科技园区创建计划，未来科技城、青山湖科技城建设取得重大进展。创建培育省市级特色小镇44个、国家级众创空间35家、国家级孵化器30家。境内外上市企业达到135家，数量居全国城市第四位；新三板挂牌企业达到347家；入选中国民营500强企业数连续14年居全国城市首位。全市人才总量达215万人，全社会研发支出与地区生产总值之比提升至3.1%左右，有效发明专利拥有量居省会城市第一，创新发展水平居全国城市前列。

　　——这五年，城乡面貌在加强统筹协调中得到大提升。我们深入实施城市国际化、城乡一体化战略，交通、电力等基础设施建设全面提速。萧山机场二期完成扩建，杭州东站综合交通枢纽和杭甬、杭宁、杭长铁路客运专线建成投用，高速公路通车里程达到632公里，市区快速路新增通车里程128公里，“四纵五横”快速路网基本形成。地铁运营里程82公里，公交出行分担率达39.8%，成为全国绿色交通城市。大力推进城乡统筹，区县协作累计到位资金20.88亿元、实施协作项目1322个；累计建成美丽乡村精品村249个、风情小镇29个、美丽乡村精品线路28条；建成省级现代农业园区85个、各级粮食生产功能区1476个。深入实施“六大西进”行动，县（市）经济发展速度加快，成为全市发展新增长点。

　　——这五年，生态环境在强化保护治理中实现大改善。我们以建设美丽中国样本为目标，坚持走“绿水青山就是金山银山”发展道路，成为省会城市中的首个国家生态市。“五水共治”取得突破，全市消灭了垃圾河、黑臭河，初步实现城区污水“零直排”、农村污水处理“全覆盖”。三堡排涝工程等一批综合治水工程建成投用。“五气共治”成效显著，率先成为无钢铁生产企业、无燃煤火电机组、基本无黄标车的“三无”城市。“五废共治”统筹推进，成为全国生活垃圾分类示范城市。累计拆除违法建筑8808.9万平方米，完成“三改”11637.5万平方米；淳安、桐庐成为全省首批基本无违建县，西湖名胜区、建德、临安、富阳成为省级无违建创建先进区县（市）。四边三化、两路两侧等专项行动深入开展，西湖、湘湖、运河、钱塘江、新安江、苕溪、西溪湿地、千岛湖等重点区域保护治理深入推进，“三江两岸”沿线生态廊道基本建成，美丽县城、风情小镇、美丽乡村串珠成为靓丽风景线。

　　——这五年，民生事业在推进共享共治中取得大成效。我们加大民生投入，办好实事项目,连续10年入选全国最具幸福感城市。城镇居民人均可支配收入从2011年的33766元提高到2016年的52185元、农村居民人均可支配收入从17086元提高到27908元，城乡收入倍差从1.98缩小到1.87；家庭人均年收入低于4600元的贫困现象全面消除，市区最低生活保障标准提高到每月819元。户籍人口期望寿命达81.85岁。累计新增城镇就业128.29万人。文化名城建设向纵深推进，浙江音乐学院、杭州国博中心建成投用，累计建成农村文化礼堂618个。大运河列入世界遗产名录，国家级非遗项目数量居同类城市第一。精神文明建设成果丰硕，“最美现象”品牌打响全国，蝉联全国文明城市称号。全国义务教育基本均衡县实现全覆盖，省基本实现教育现代化区县（市）达到9个；幼儿教育普惠发展，高中教育普及发展，高等教育和职业教育提升发展。健康杭州建设持续深化,公立医院改革、医疗改革、医养护签约服务工作走在全国前列，5所市属综合性医院建成投用。基层社会治理不断加强，获得全国平安综治优秀市、和谐社区建设示范城市称号。

　　——这五年，政府治理能力在加强自身建设中取得大提高。我们扎实开展党的群众路线教育实践活动、“三严三实”专题教育和“两学一做”学习教育，严格落实中央八项规定精神，狠抓干部能力作风建设。市政府坚持重大问题向市委汇报，主动接受人大和政协的监督，五年累计办理市人大代表建议2981件、政协提案2896件；提请审议地方性法规草案37件，制定修改政府规章61件。建立了政府常务会议学法制度，推行政府法律顾问全覆盖。完成新一轮政府职能转变和机构改革。深入推进“四张清单一张网”改革。启动规范化绩效管理，实行重大民生决策公众咨询监督制度，完善公述民评等问政监督渠道，社会共治进一步强化。预决算公开有序推进，财政透明度显著提升。认真落实全面从严治党要求，坚决开展反腐败斗争，坚决纠正“四风”，严格正风肃纪，营造勤政廉洁、风清气正的政务环境。

　　各位代表！上述成绩的取得，是党中央国务院亲切关怀、省委省政府和市委坚强领导的结果，是全市广大干部群众凝心聚力、顽强拼搏、团结奋斗的结果。每一个杭州人，都是这段光荣历程的亲历者、见证者、推动者。在此，我代表市人民政府，向全市人民和外来建设者，向人大代表、政协委员，向各民主党派、工商联、无党派人士、各人民团体和各界人士，向离退休老同志，向中央在杭单位、各类驻杭机构和省级各单位，向驻杭人民解放军、武警官兵，向所有参与和关心支持杭州建设发展的港澳台同胞、海外华人华侨、国际友人和各界朋友，表示衷心的感谢和崇高的敬意！

　　我们也清醒认识到，杭州发展中的问题和挑战。主要有：供给侧结构性改革需要进一步深化，科技创新能力有待增强，新旧发展动能转换还需付出更大努力；城市国际化水平还不够高，市民文明素质仍需提升，城市治理能力有待提高；生态质量有待进一步优化提升，特别是空气、水体、土壤等环境治理任务繁重；改善民生任务较为艰巨，城乡居民持续增收压力加大，公共服务、安全稳定等领域还存在很多短板，离人民群众的要求还有较大差距。同时，一些部门及工作人员的理念、能力、作风与形势发展还不相适应，负责担当意识有待进一步增强，消极腐败问题在一些地方和领域仍然存在。对此，我们一定采取有力措施，努力加以解决。

**二、今后五年发展目标任务**

　　各位代表！杭州已站在新的历史起点上，习近平总书记在G20杭州峰会期间提出“秉持浙江精神，干在实处、走在前列、勇立潮头”的新要求，赋予我们新的使命和责任。我们必须认真贯彻落实杭州市第十二次党代会精神，准确把握杭州发展的战略定位、主要目标和总体部署。杭州未来发展战略定位是：加快城市国际化，建设独特韵味别样精彩的世界名城。今后五年主要目标任务是：全面完成“十三五”规划任务，确保亚运会筹备工作基本就绪，率先高水平全面建成小康社会，实现国际化水平、综合实力、治理现代化水平、人民生活品质显著提升，努力成为具有较高全球知名度的国际城市。

　　面对杭州发展新方位、新使命、新目标，今后五年政府工作总体要求是：高举中国特色社会主义伟大旗帜，深入学习贯彻习近平总书记系列重要讲话精神和治国理政新理念新思想新战略，按照“五位一体”总体布局和“四个全面”战略布局，全面贯彻落实新发展理念，坚持稳中求进工作总基调，以提高发展质量和效益为中心，以城市国际化为主抓手，强化改革创新、扩大开放合作、提升环境品质、保障改善民生，不断厚植创新活力之城、历史文化名城、生态文明之都、东方品质之城的特色和优势，当好全省干在实处走在前列勇立潮头的排头兵，为建设独特韵味别样精彩的世界名城打下坚实基础。

　　实现今后五年目标任务，必须统筹谋划落实重大举措，尤其要用好“后峰会、前亚运”等战略机遇，在扬长补短、彰显特色上下功夫，进一步把特色优势转化为竞争优势、发展胜势，加快杭州经济社会在高起点上的跨越发展。

　　第一，着力厚植创新创业的特色和优势。创新创业是杭州发展的活力所在。必须深入实施创新驱动发展战略，加快国家自主创新示范区建设，努力使杭州成为具有全球影响力的“互联网+”创新创业中心。深入实施人才强市战略，构建更具竞争力的人才集聚机制、充满活力的人才使用机制、与时俱进的人才评价机制，进一步补齐居住、教育、医疗等人才保障短板，吸引更多人才来杭创新创业。到2021年人才总量达到275万人以上，高技能人才占技能劳动者比例超过30%。深入构建全要素创新生态系统，大力弘扬创新文化，打通科技和经济转移转化的通道，让各类创新要素释放更大能量。到2021年，全社会研发支出与全市生产总值之比达到3.5%。持续深化商事制度改革，创造更优的营商环境。坚持以科技创新为引领，大力鼓励发展特色小镇、双创基地和众创空间，争取国家实验室、国家大科学中心和重大科技基础设施落地，促进更多的创新主体和创新项目集聚。加快城西科创大走廊、城东智造大走廊、钱塘江生态经济带、运河湖滨高端商务带、钱塘江金融港湾等重大平台建设，拓展发展新空间。深入实施“一号工程”，做强战略性新兴产业，提升传统优势产业，振兴实体经济，做大集成电路、高端装备制造、新能源汽车、物联网、机器人、生物医药、新材料等重点产业，培育人工智能、量子通信、新型显示、虚拟现实等未来产业，抢占发展制高点。充分发挥民营经济优势，加快民营经济转型发展。壮大主导和支柱产业规模，形成万亿级信息产业集群和更多千亿规模产业；加强企业梯队建设，着力培育世界级企业、千亿级企业和一大批创新型领军企业。

　　第二，着力增强文化引领的特色和优势。文化是城市可持续发展的源动力。必须坚定文化自信自觉，加强历史文化传承和现代文化发展，更好发挥文化在引领风尚、教育人民、服务社会、推动发展等方面的作用。大力弘扬中华优秀传统文化，深入挖掘良渚文化、吴越文化、南宋文化等传统文化资源，加大物质和非物质文化遗产保护利用力度，打响丝绸、茶叶、中医药、瓷器、书画等特色品牌，进一步彰显历史文化魅力。发挥大运河、西湖两大世界遗产综合效应，争取良渚古城遗址申遗成功，推动南宋皇城遗址等申遗工作，打造世界遗产群落。扩大对外人文交流，展示东方文化特色，建设东方文化国际交流重要城市。加强城乡建筑、设施、景观设计和色彩管理，增强地域文化特色，提升城乡文化品位和文化价值。顺应文化与科技、经济渗透融合趋势，积极发展演艺等特色产业，建设具有较高国际知名度的文化创意中心。深入开展社会主义精神文明建设，大力弘扬“精致和谐、大气开放”的城市人文精神和G20杭州峰会服务精神，进一步打响“最美现象”品牌，加强市民科学、道德和人文素养教育，持续提升市民文明素质和城市文明程度。

　　第三，着力增创城乡协调发展的特色和优势。城市国际化与城乡一体化互动融合发展，是杭州城市独特魅力所在。必须加快构建区域协同发展新格局，争创国家中心城市。树立“精明增长、紧凑城市”理念，落实“多规合一”要求，完成新一轮《杭州市城市总体规划》修编，构建多中心、组团式、网络化、生态型、一体化的市域空间布局。实施钱塘江两岸规划优化，充分发挥两岸建设对杭州城市发展的战略支撑作用。优化城市人口和功能布局，控制主城区人口增量，推进主城区非核心功能疏解。加快钱江新城二期、钱江世纪城、大江东新城、未来科技城、青山湖科技城、之江新城以及大城北地区等重点区域开发建设。持续推进城市有机更新，全面完成主城区城中村改造和小城镇环境综合整治任务，加快完成城乡危旧房治理和老旧住宅小区综合整治，实现无违建区县（市）全覆盖。加强城市防洪排涝规划建设、地下空间开发和综合管廊建设，提高城市承载保障能力。深入实施新一轮城乡区域统筹发展政策，加大要素转移和产业帮扶力度，加强农村道路提升改造，加快中心镇、特色镇和美丽乡村建设，促进城乡高质量、均衡性发展。实施萧山国际机场打造长三角核心机场行动计划，争创国家级临空经济示范区，建设亚太地区重要国际门户枢纽。建成杭黄铁路，加快建设沪乍杭、商合杭、杭温、杭绍台铁路，推进杭州“一轴两翼五站”铁路枢纽布局建设，构建全国重要高速铁路枢纽城市。到2022年确保建成10条地铁线和2条城际铁路线,运营里程达到446公里，成为国内一流“地铁城市”。加快城市组团环线和交通廊道建设，基本建成市域800公里高速公路、市区464公里城市快速路网，构建高效交通组织系统。

　　第四，着力拓展开放合作的特色和优势。按照建设融合线上线下、联通世界、服务全球的国际重要商贸中心要求，积极用好国际国内两个市场、两种资源，扩大开放、深化合作、拓展优势。主动对接“一带一路”倡议，更好融入长江经济带和长三角城市群发展，加快杭州都市区建设，提升杭州都市圈协同发展水平。深入实施浙商回归工程，高质量引进外资项目，支持企业“走出去”，推动招商引资新发展。深化跨境电商综试区建设，构建全球最优跨境电商生态圈，全力推进全球电子商务平台建设，打造“网上丝绸之路”重要战略枢纽城市。推进国家下一代互联网示范城市和5G应用先行区建设，努力成为大数据开发应用重要示范城市。深化会展管理体制改革，引进高端国际会议展览项目和国际组织，打造国际会议目的地城市和会展之都。实施新一轮旅游国际化和旅游全域化行动计划，大力发展乡村旅游，建设国际重要的旅游休闲中心。积极引进国际性赛事项目，打造国际赛事之城；努力承办好第13届全国学生运动会、第14届世界短池游泳锦标赛及世界游泳大会，推进亚运村和场馆设施配套规划建设、高水平筹办2022年第19届亚运会。

　　第五，着力彰显生态良好的特色和优势。坚持“绿水青山就是金山银山”的发展思想，强化生态环境保护治理。统筹实施生态保护修复工程，加强城市西部生态保护，加快建设城市组团间的生态廊道，构筑大杭州生态安全屏障。牢固树立生态红线理念，划定城镇开发边界、永久基本农田和生态保护红线，坚决守住开发强度警戒线。深入推进水体、空气、固废、土壤等突出环境问题治理，加强污水和固废处置设施规划建设，增强环境承载能力。加大产业结构和能源结构调整力度，落实节能减排降耗综合措施，全市域建成清洁排放区。加强环保督察，深化区域联防联控，强化生态环保制度建设，零容忍打击各类违法行为，决不允许把污染成本转嫁给社会。确保生态环境质量指数居全国大中城市前列，努力建设颜值高、气质好的生态文明之都。

　　第六，着力提升宜居宜业的特色和优势。完善就业创业支撑体系，提高全社会就业水平，多途径增加城乡居民收入。实施全民参保计划，完善社会保障和社会救助体系，基本实现人人享有社会保障。全面推进教育现代化，增强教育对经济社会发展的支撑作用。推进十五年基础教育高水平、均衡化发展，提升发展职业教育，加快发展地方高等教育。实施《“健康杭州2030”规划纲要》，扩大优质卫生和健康服务供给。深化国家和省级养老服务业综合改革试点，健全养老服务体系。促进妇女儿童事业健康发展。强化食品药品安全管理。推进社会诚信体系建设，打响“诚信杭州”品牌。推动政府数据开放，拓展智慧应用的深度和广度。加强城市管理，提升标准化、精细化管理水平。优化公交网络布局，完善公共交通体系。深化网格化管理，加强社会治安和生产安全、公共安全、金融安全管理，健全防灾减灾和突发事件应急处置机制。我们要把以人民为中心的发展思想，落实到加快实现公共服务优质均衡、人民生活幸福和谐、社会公平正义的行动上，努力建设共治共管、共建共享的东方品质之城。

　　各位代表！我们要抓住机遇、增创优势，落实举措、开创未来，一定要让杭州经济更发达、城乡更美丽、生态更良好、生活更舒适、社会更和谐。

**三、2017年重点工作安排**

　　2017年是新一届政府的开局之年，也是实施“十三五”规划的重要一年。我们要着眼五年、立足当年，围绕大局、统筹兼顾，突出重点、把握关键，开拓创新、抓实工作，促进经济持续健康发展和社会和谐稳定，努力开创杭州改革发展新局面。

　　今年发展主要预期目标是：地区生产总值增长8%左右，一般公共预算收入增长7.5%，城镇和农村居民年人均可支配收入分别增长8%左右；全面完成省下达的节能减排任务。

　　重点抓好七个方面工作：

　　（一）加快推进重点领域改革，着力增强体制机制活力。推进“最多跑一次”改革。完善“四张清单一张网”，深化“放管服”改革，全面推行“互联网+政务服务”，普及网上办事，确保到年底“最多跑一次”改革覆盖80%的行政权力事项。实现行政许可标准化建设、工程建设项目“多测合一”等全市域覆盖，进一步提升项目审批速度。全面开展“双随机、一公开”监管。深化行政管理体制改革。推进综合执法体制改革。深化大交通体制改革。加强出租车行业规范化管理。推行新型居住证制度，实施积分制管理。落实环保机构监测监察执法垂直管理制度改革工作。深化信用杭州建设。设立政府大数据管理机构。扎实推进萧山、余杭、富阳等与主城区公共服务一体化。加强中心镇和小城市培育试点镇改革发展。深化投融资体制和要素配置方式改革。深入推进国企分类改革，大力发展混合所有制经济。做优做强国有投资平台，发挥各类政府投资基金引导和放大作用。盘活利用批而未供土地2.5万亩，开发建设供而未用土地4.5万亩，完成低效用地再开发3万亩，垦造水田9750亩以上。完善工业用地弹性出让制度，深化“零地技改”和亩产效益综合评价。大力支持私募金融和创新基金健康发展，推进互联网金融规范化发展，提升金融服务实体经济能力。深化“三去一降”工作。淘汰落后产能100家（项）以上，整治提升“低小散”企业300家以上。坚持“房子是用来住的、不是用来炒的”定位，进一步加强和改进房地产调控，确保房地产市场稳定健康发展。争取“债转股”试点，防范金融风险。加大减税清费力度，进一步降低企业特别是小微企业的负担。

　　（二）加快推进产业转型升级，着力提升实体经济质量。优化投资结构。完成固定资产投资6135亿元，增长5%；确保重大基础设施、重大产业项目、重大技术改造、生态环保投资增长15%以上。力争浙商回归到位资金722亿元，实际利用外资65亿美元，内资到位资金1540亿元。加快发展新兴产业。深入推进信息经济“六大中心”建设，加快人工智能、虚拟现实、量子通信、物联网等应用发展。加快集成电路、网络设备、智能终端等信息制造业和高端制造业发展，积极培育航空航天产业，加快军民融合发展。争创“中国制造2025”试点示范城市。提升改造传统产业。加大工业技改力度，推进“两化”深度融合，深入实施“互联网+”“标准化+”“品牌+”“机器人+”等行动，启动万企转型升级和企业上云计划，推动优势传统产业转型升级。加强本土跨国公司和大企业培育，加快企业股份制改造。滚动实施小微企业三年成长计划，全市新增“个转企”2500家以上。支持建筑业提升发展。加快发展服务业。做强现代物流、科技、会展等生产性服务业，做优养老、健康、体育等生活性服务业。打造旅游休闲百强示范项目，创建国家全域旅游示范区。推动15家省级服务业集聚区提升发展。实施“放心消费在杭州”行动计划，提升一批商业中心和特色街区，确保社会消费品零售总额增长10%。促进农业农村发展。推进农业供给侧结构性改革，实施“整洁田园、美丽农业”行动。完善农业服务体系，加大科技兴农力度，发展多种形式农业规模经营，提高农业组织化程度。加快培育新型职业农民和现代农业经营主体，做大农村电商、乡村旅游、民宿等新业态，推进农村一二三产融合发展，促进农民持续增收。深化村（社区）经济合作社股份制改革。采取切实措施，千方百计发展村级集体经济，打赢消除集体经济薄弱村攻坚战。深化美丽乡村建设，推广杭派民居，打造美丽乡村升级版，新增建设7个风情小镇、20个3A级村落景区。

　　（三）加快推进发展动能转换，着力提升创新驱动能力。深化创新改革试点。落实杭州国家自主创新示范区发展规划和空间布局规划，更好发挥示范区创新引领作用。深入实施“创新创业新天堂行动”，深化小微企业创业创新基地城市示范建设。加快省级全面创新改革试验区建设，争创国家全面创新改革试验区。加强重点平台建设。支持大江东产业集聚区创新发展、支持杭州高新区建设世界一流高科技园区、支持杭州经济开发区提升发展，充分发挥国家级和省级开发区的作用。加强城西科创大走廊基础配套建设，进一步优化创新生态体系；加快城东智造大走廊规划建设。坚持以特色小镇建设理念改造传统工业园区，加快提升工业园区（开发区）整体发展水平。聚焦金融特色小镇和金融集聚区发展，筹建钱塘江金融港湾展示服务中心。创建省级高新技术特色小镇，打造一批行业示范和创新标杆特色小镇。建设一批市级双创示范基地，新增市级众创空间20家以上、市级孵化器12家以上、孵化面积12万平方米。推动创新政策落地。落实高校院所和科技中介技术交易额补助政策，促进科技成果转化。设立杭州市跨境风险投资引导基金，推进科技金融结合试点城市建设，争创国家投贷联动试点城市。组织重大科技创新项目25项以上，有效发明专利达到3.6万件以上；争取全社会研发支出与地区生产总值之比达到3.2%。加强创新企业培育。积极支持中小企业特别是科技型企业发展，新增国家重点扶持高新技术企业340家以上、省科技型中小企业1370家以上、市重点培育科技型初创企业300家以上。新增省级重点企业研究院、省级企业研究院和省级研发中心80家。加强人才培育和引进。深入实施国家和省“千人计划”、市“521”全球引才计划、“115”海外引智计划，放大“一会一赛”品牌效应，全年新引进和培育各类人才10万名。加大高技能人才培育力度。加强企业经营管理人才队伍建设，提振企业家信心，进一步激发发展动能。

　　（四）加大内外开放力度，着力拓展合作发展空间。提升国际服务功能。实施2017年城市国际化行动方案和项目计划。完善国际化标识系统，优化多语种服务综合平台。加快推进教育医疗卫生等重点领域公共服务国际化，加强街区、社区、楼宇等领域的国际标准化建设。推进杭州国家互联网交换中心试点和通信骨干直连点建设。加快城市国际化促进条例立法工作。推进门户枢纽项目建设。加快萧山机场集疏运体系建设，完善机场基础设施，开辟更多国际国内客货运航线。优化完善杭州铁路枢纽，推进城西综合交通枢纽规划建设。加快京杭运河“四改三”工程杭州段和二通道工程建设，推进海河联运和“散改集”项目试点。加快对外经贸转型发展。积极支持跨境电商、外贸综合服务等外贸新业态发展，大力培育外贸新动能。深化服务贸易创新发展试点建设，推动外贸方式转变。建设跨境电商大数据平台，提升跨境电商产业园区服务能力。支持企业“走出去”，实现创新链产业链价值链全球化布局，构建开放型经济新格局。推进都市区建设和都市圈协同发展。深化区域合作，加强杭州都市区和都市圈基础设施对接配套、产业融合发展、公共服务共建共享，更好发挥杭州中心城市的集聚辐射作用。切实做好对口支援和扶贫协作等工作。

　　（五）加大统筹建设力度，着力提升城乡功能品质。加强重大规划编制管理。进一步落实市区规划统一管理制度，编制分区规划，编制钱塘江两岸综合保护、亚运会场馆和亚运村等重大专项规划。研究制定主城区非核心功能疏解规划。加快交通道路等重大基础设施建设。加快推进地铁二期工程建设，新增地铁运营里程35公里。全面开工建设地铁三期工程。加快杭富、杭临等城际铁路建设。启用杭州铁路南站。推进杭州绕城高速西复线、千黄高速、杭宁高速改扩建、临金高速、杭甬高速复线杭州段、“杭州中环”（城市组团环线）等重大交通项目建设，建成杭金衢高速公路拓宽工程。加快“四纵五横”和“三连十一延”快速路网建设，开工建设艮山东路提升改造、文一西路快速路西延、江南大道快速路等工程；加快完成天目山路-环城北路快速路、坎红路-红十五线快速路、留祥路西延等工程技术前期，力争早日开工建设。加快望江隧道等过江通道建设。建成地下综合管廊试点30公里，开发利用地下空间500万平方米。加快推进治危拆违、城中村改造和小城镇环境综合整治。完成“三改”1200万平方米，拆除违法建筑1400万平方米，力争50%以上区县（市）完成省级基本无违建县（市、区）创建。深化征地拆迁和回迁安置“清零”专项行动。完成地质灾害隐患点综合治理313个以上，新建和改扩建避灾安置点220个。加快城中村改造，确保主城区全面完成改造22个村以上、基本完成改造28个村以上，加强城中村拆后空间的规划利用。全面推进149个小城镇环境综合整治，确保30%的乡镇（街道）通过省级考核。提高城乡综合管理水平。以智慧政务为核心，推动各领域智慧应用。深化乡镇（街道）“四个平台”建设，加强农村社会治理，加强撤村建居社区管理，完善城乡社区协商共治机制，提高社区治理水平。加强城市管理，提升洁化序化亮化绿化美化水平。加强共享单车综合管理。切实加强农村建房规范管理，完善村庄环境管理长效机制。

　　（六）加大环境治理力度，着力提升生态建设水平。打赢劣V类水剿灭战。实施清水入城、截污纳管、雨污分流、大市政管网配套及泵站建设等四个行动计划，确保年底消除劣V类水体；加快建设七格四期、城西、之江等污水处理设施和管网，新增城镇污水配套管网150公里；全面完成规模畜禽养殖场（户）整治，加强建制村生活污水处理设施长效运营维护工作；完成14座病险水库除险加固和80座山塘整治，建设萧围北线标准塘、大江东片外排工程；推进千岛湖配供水工程，开展老旧供水管网改造，深化节水工作；完成70个海绵城市建设项目。持续深化大气污染治理。以治霾为中心，全面完成小燃煤锅（窑）炉淘汰和热电锅炉清洁化改造，关停转迁一批废气污染严重的工业污染源，启动清洁排放区建设。持续推进重点行业挥发性有机物专项整治，继续淘汰更新老旧车辆。建设500个公用充电桩（枪），1000个自用和共用充电桩（枪）。大力提升固废处置能力。加快临江、七格、天子岭等污泥、建筑固废和生活垃圾处置设施建设，推进城西、之江、城东和萧山等分类减量综合体建设，九峰环境能源项目投入运行。扩大智慧垃圾分类试点，有效实现生活垃圾减量化。进一步加强工业固废、医疗固废处置能力建设。加快能源资源节约利用。推进钱江变、机场变、庆隆变等能源基础设施项目建设，完善市域电力设施布局。加大工业节能力度。开展土壤环境治理行动。推进建筑节能改造，提高装配式建筑占比。

　　（七）加大民生保障力度，着力促进社会共建共享。多渠道促进居民增收。完善鼓励创业政策体系，新增城镇就业21万人。实施精准扶贫，推进低收入群体增收，多途径增加城乡居民收入。深化“春风行动”，完善困难帮扶救助政策，健全社会救助体系。推动教育优质均衡发展。加快推进新名校集团化战略，加强义务教育配套学校建设，提高基础教育办学水平。实施学前教育、特殊教育、民办教育和终身教育发展新政，深化职业教育质量提升工程，推进民办西湖大学等新型高校建设，支持杭师大等市属高校提升办学水平。提升卫生和计生服务水平。推动医养护签约服务提质扩面，加快分级诊疗制度建设。深化智慧医疗惠民服务，加大优质医疗资源“双下沉、两提升”力度。完成全国第三批公立医院综合改革试点任务。推进萧山等4个先行先试地区综合医改工作。全面实行生育登记服务制度。促进养老健康产业发展。高度重视老龄化问题，完善相关政策制度，推进养老服务业综合改革试点。推进国家医养结合试点建设、社会保险制度改革工作。推动“智慧社保”，实施基层社会保险工作标准化国家试点。加大对失独家庭帮扶力度。加强城乡公共无障碍设施建设，加强对残疾人的关爱。推进公共文化体育设施建设。建设良渚文化国家公园（一期）项目。建成G20杭州峰会博物馆，推进城市档案中心、之江文化中心等重大文化项目建设。新建农村文化礼堂100座。社区（村）公共文化场所无线网络覆盖新增1000家。新建城乡公共体育设施20处。推进全民健身实施计划，办好全国学生运动会。加强城市安全管理。落实安全生产责任制，健全防灾减灾、应急救援体系，完善食品药品监管体系，推进国家食品安全示范城市创建。全面建设网络维权国家“两中心”。深化“平安杭州”建设，加强社会治安综合治理，强化网络信息安全管理，确保社会平安稳定。

　　认真办好十个方面民生实事。①持续深化治水治气。完成河道清淤综合整治245公里，雨污分流截污纳管项目200个，清洁化改造热电锅炉27台，新增太阳能光伏发电装机容量100兆瓦以上，回收汽油1000吨以上。②持续改善交通出行。主城区新建停车泊位4.5万个以上，新增和改建公共自行车服务点120处，更新公共自行车1万辆；新辟和优化公交线路40条。③提升城乡生活环境。新增市区绿地400万平方米；新增管道燃气用户5万户；新增垃圾分类清运集置点规范设置100处，分类垃圾房提升改造70座；改造50条城市道路路灯；完成公厕提升改造70座。④完善社会抚养体系。新改扩建50个街道（乡镇）级综合性居家养老服务照料中心；全面实施孤儿及困难家庭儿童生活费和补贴发放政策。⑤强化食品药品安全治理。新创建 “三净四无五可”标准餐饮安全示范店150家，“六有三无”标准诚信示范药店100家。⑥强化电梯安全监管。建立电梯维保信息化平台；开展老旧小区电梯安全隐患消除工作和老旧住宅增设电梯试点工作。⑦加强居民住房保障。新开工保障性住房4.6万套，竣工4.5万套；农村住房改造1.2万户、危房改造1210户；新推出公共租赁住房（廉租住房）6000套，货币补贴1500户；治理城镇危旧房196幢、18.1万平方米。⑧优化扶持创新创业。新增小微创业园5家，资助大学生创业项目200家。⑨优化医疗保障服务。县级以上医院及主城区社区卫生服务机构全面推广“医信付”，让群众享受“先诊疗后还款”服务。⑩保障菜篮子产品供应。新建菜篮子基地5000亩，蔬菜保险覆盖面积5000亩，向全市直供直销24万吨以上；地产农产品质量抽检合格率达98%以上。

**四、切实加强政府自身建设**

　　完成各项目标任务，对新一届政府自身建设提出了更高要求。我们要坚持党的领导，进一步加强法治政府、服务政府和廉洁政府建设，不断提高政府治理现代化水平，努力建设人民满意政府。

　　（一）忠诚坚定、从严治政，坚持党的领导。牢固树立和切实增强政治意识、大局意识、核心意识、看齐意识，高度自觉地在思想上政治上行动上同以习近平同志为核心的党中央保持一致，坚决维护党中央权威。加强思想政治建设，严守政治纪律和政治规矩，切实做到忠诚干净担当。坚决贯彻落实党中央国务院、省委省政府和市委各项决策部署，在市委的领导下开展工作。坚决把全面从严治党要求落实到从严治政之中，落实到政府公务人员能力和作风建设之中，努力打造勇立潮头浙江铁军的排头兵。

　　（二）尊崇法治、依法行政，建设法治政府。切实将政府活动全面纳入法治轨道，严格按程序决策、按规矩办事。依法接受市人大及其常委会的工作监督和法律监督，认真落实各项决议决定并定期报告工作。自觉接受市政协的民主监督，认真听取各民主党派、工商联、无党派人士和人民团体的意见。认真办理人大代表议案建议和政协提案，进一步提高办理质量。自觉接受监察监督，主动接受社会和舆论监督。加强重大行政决策、规范性文件合法性审查，全面落实政府法律顾问制度，加强政府参事工作，鼓励公民参与政策制定和社会治理，提高决策科学化、民主化、法治化水平。落实重大行政决策程序，严格执行行政执法责任制，规范行政执法行为，让权力公开透明、阳光运行。

　　（三）务实创新、高效施政，建设服务政府。“政者，口言之，身必行之”。政府及其工作人员要强化负责担当精神、提升创新实干能力，在落细落小落实中谋大事、干大事、成大事。要坚持实干至上、行动至上，说了就要干、定了就要干，做到重大决策和重点工作立说立行、一抓到底。全面推行政府工作项目化，对企业和群众承诺的事，要马上就办、说到做到，对企业和群众关心的事，要换位思考，当好“店小二”。真正以人民群众满意度作为评判工作的标准，推进行政服务规范化和标准化建设，完善便民服务体系，加强政府信息公开，努力让群众对政府工作过程和结果双满意。加强绩效管理考核，完善常态化督促检查工作机制和约束机制，确保重大决策部署落实到位。

　　（四）干净自律、勤勉为政，建设廉洁政府。严格执行党内政治生活若干准则、纪律处分条例、党内监督条例、问责条例、廉洁自律准则，认真落实党风廉政建设主体责任和“一岗双责”。严格贯彻落实中央八项规定精神，持之以恒反“四风”，始终保持廉洁本色。强化督查问责，严厉整肃庸政懒政怠政行为。坚持政府过紧日子，集中财力办大事，完善财政预算管理，加强政府债务管理。深入推进审计监督，加大公共资金、国有资产、国有资源监管力度。强化对重点领域和关键岗位的监督，坚决查处不正之风和腐败行为，切实做到政治清明、政府清廉、干部清正。

　　各位代表！杭州改革发展正处在关键时期，任务艰巨，责任重大。让我们紧密团结在以习近平同志为核心的党中央周围，在省委省政府和市委的领导下，团结拼搏、扎实工作，全力推动杭州率先高水平全面建成小康社会，加快推进杭州世界名城建设，以优异成绩迎接党的十九大胜利召开！
